# Severe Retroperitoneal Hemorrhage in a COVID-19 Patient on a Therapeutic Dose of Low Molecular Weight Heparin: A Case Report

**DOI:** 10.7759/cureus.26275

**Published:** 2022-06-24

**Authors:** Martin Dubovský, Marianna Hajská, Arpád Panyko, Marián Vician

**Affiliations:** 1 Surgery, Faculty of Medicine, Comenius University Bratislava, Bratislava, SVK; 2 IVth Department of Surgery, University Hospital Bratislava, Bratislava, SVK

**Keywords:** retroperitoneal hemorrhage, spontaneous bleeding, lmwh overdose, anticoagulant therapy, covid-19

## Abstract

Extensive drug treatment for coronavirus disease 2019 (COVID-19) includes low molecular weight heparin (LMWH). At therapeutic doses of LMWH, there is an increased risk of bleeding complications. Spontaneous, non-traumatic bleeding into the retroperitoneum is a life-threatening condition that can progress very rapidly. We describe a complication of COVID-19 bronchopneumonia treatment in which a patient developed a shock condition caused by non-traumatic bleeding into the retroperitoneum and abdominal wall due to LMWH overdose. The patient was operated on under difficult conditions - in biosafety level 3 (BSL-3). This case is exceptionally fascinating and informative. Nowadays, it is essential to point out possible complications associated with the treatment of COVID-19. Based on this report, we emphasize the need for careful LMWH dosing and quick and accurate diagnosis. Surgeons should maintain a higher index of suspicion for spontaneous bleeding in non-specific abdominal pain patients with COVID-19 or patients receiving therapeutic doses of LMWH.

## Introduction

The coronavirus disease 2019 (COVID-19) has been associated with thrombosis as one of the disease's complications [1]. Once patients are hospitalized with COVID-19, they usually receive treatment accompanied by anticoagulants to prevent thrombosis [2]. The drug name, dosage, or duration is not yet strictly defined, as there is still a lack of guidelines in this matter. The thromboembolic pathologies and mortality have increased in individuals infected with COVID-19 [3]. Clinical studies [4-6] already in the first pandemic period have reported that COVID-19 patients are predisposed to thromboembolism.

On the other hand, Tanal et al.’s [2] findings of non-traumatic focused hemorrhages are unexpected. Several clinical reports [7-12] describing cases of severe spontaneous abdominal or retroperitoneal bleeding were published during the most fulminant phase of the pandemic. Retroperitoneal hematoma is a rare radiologically diagnosed complication, defined as bleeding in the retroperitoneal space, usually without associated trauma or iatrogenic manipulation. It is generally seen in patients receiving systemic anticoagulation including warfarin [13]. Other than anticoagulant therapies and clotting disorders, it has been associated with hematologic diseases, malignancies, trauma, and Evans syndrome [14]. Compared to other areas of bleeding, as Tanal et al. [2] show, retroperitoneal hematoma diagnosis can be challenging due to asymptomatic or non-specifically symptomatic conditions. Its treatment can be even more challenging because other comorbidities usually should be taken care of [2].

## Case presentation

A 43-year-old patient was admitted to the intensive care unit (ICU) of the Pneumology Department on December 28, 2020, because of bilateral COVID-19 bronchopneumonia. According to the chest X-ray, bilateral inflammatory infiltrates were present. The blood test showed dehydration, severe hypoxemia, hypocapnia, and elevated inflammation.

We initiated high-flow nasal oxygen (HFNO - 40/40) therapy treatment, intravenous antibiotic therapy (ceftriaxone, moxifloxacin, fluconazole), systematic corticoids (dexamethasone), and low molecular weight heparin (LMWH) in the full anticoagulation dose (Clexane, 2 x 0.8 mL subcutaneously). We also started regular antiviral therapy during the treatment (acidum ascorbicum, vigantol, cetirizine, famotidine).

From his personal history from 2013, we knew that he underwent bilateral transplantation of lungs because of idiopathic pulmonary arterial hypertension. The patient's therapy was also consulted with the transplant center. Standard post-transplant treatment with tacrolimus for immunosuppression at 2.5 mg/24 hours was continued. The patient's clinical state and respiratory parameters gradually improved.

From January 5, 2021, the patient complained about experiencing stomach pain within the hypogastrium, bilaterally, and more on his left side. The symptoms and pain worsened with time. During the ultrasound examination of the abdomen, the fluid formations were found. One fluid formation of the size 120 x 100 x 80 mm was located in left mesohypogastrium, and other two fluid formations were located in the left retroperitoneum and the right musculus rectus abdominis. Final evaluation of the ultrasound examination suspected hematomas.

**Figure 1:**
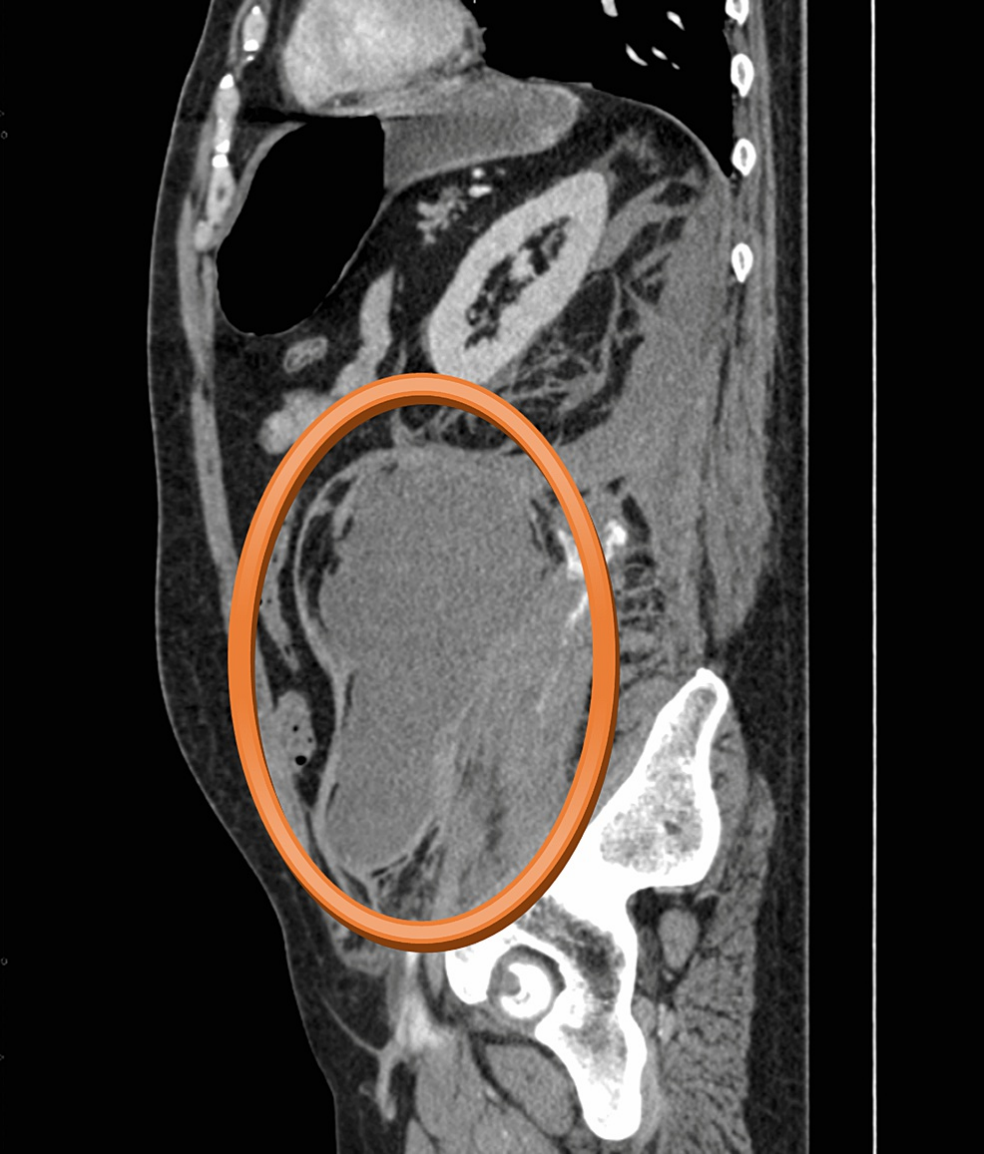
CT scan - sagittal plane The hematoma in the left retroperitoneum was of size 170 x 125 x 120 mm, attached to the left musculus psoas major, with present active bleeding

**Figure 2:**
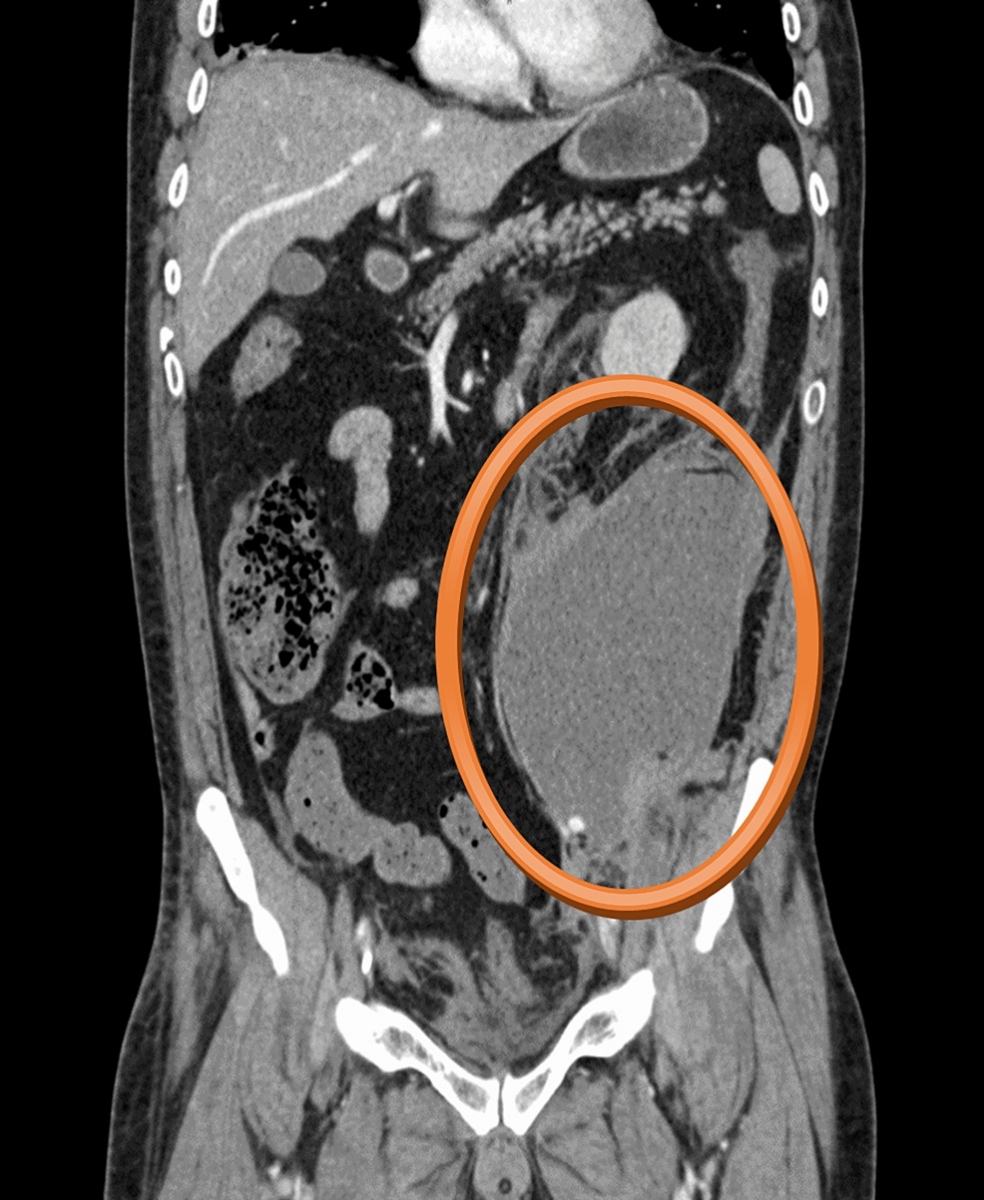
CT scan - coronal plane The hematoma in the left retroperitoneum was of size 170 x 125 x 120 mm, attached to the left musculus psoas major, with present active bleeding

**Figure 3:**
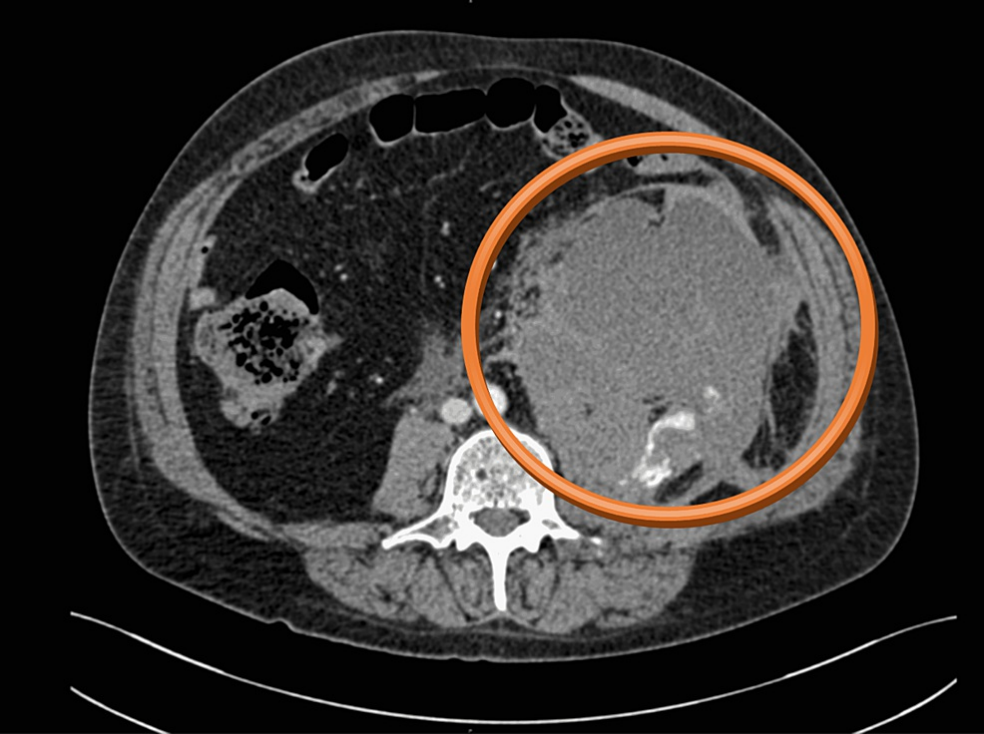
CT scan - axial plane The hematoma in the left retroperitoneum was of size 170 x 125 x 120 mm, attached to the left musculus psoas major, with present active bleeding

The patient was hemodynamically unstable. Blood test displayed a decreased hemoglobin level of 90 g/L, Leu of 28 x 10^9^/L, INR of 1.15, APTT of 1.02 seconds, fibrinogen of 2.33 g/L, hypotension (87/55 mm Hg), heart rate of 112 beats per minute. Computed tomography (CT) of the abdomen and small pelvis revealed active bleeding in the abdominal cavity, left retroperitoneum, and right musculus rectus abdominis. The left retroperitoneum hematoma was 170 x 125 x 120 mm, attached to the left musculus psoas major, and presented with active bleeding. (Figures 1-3).

**Figure 4:**
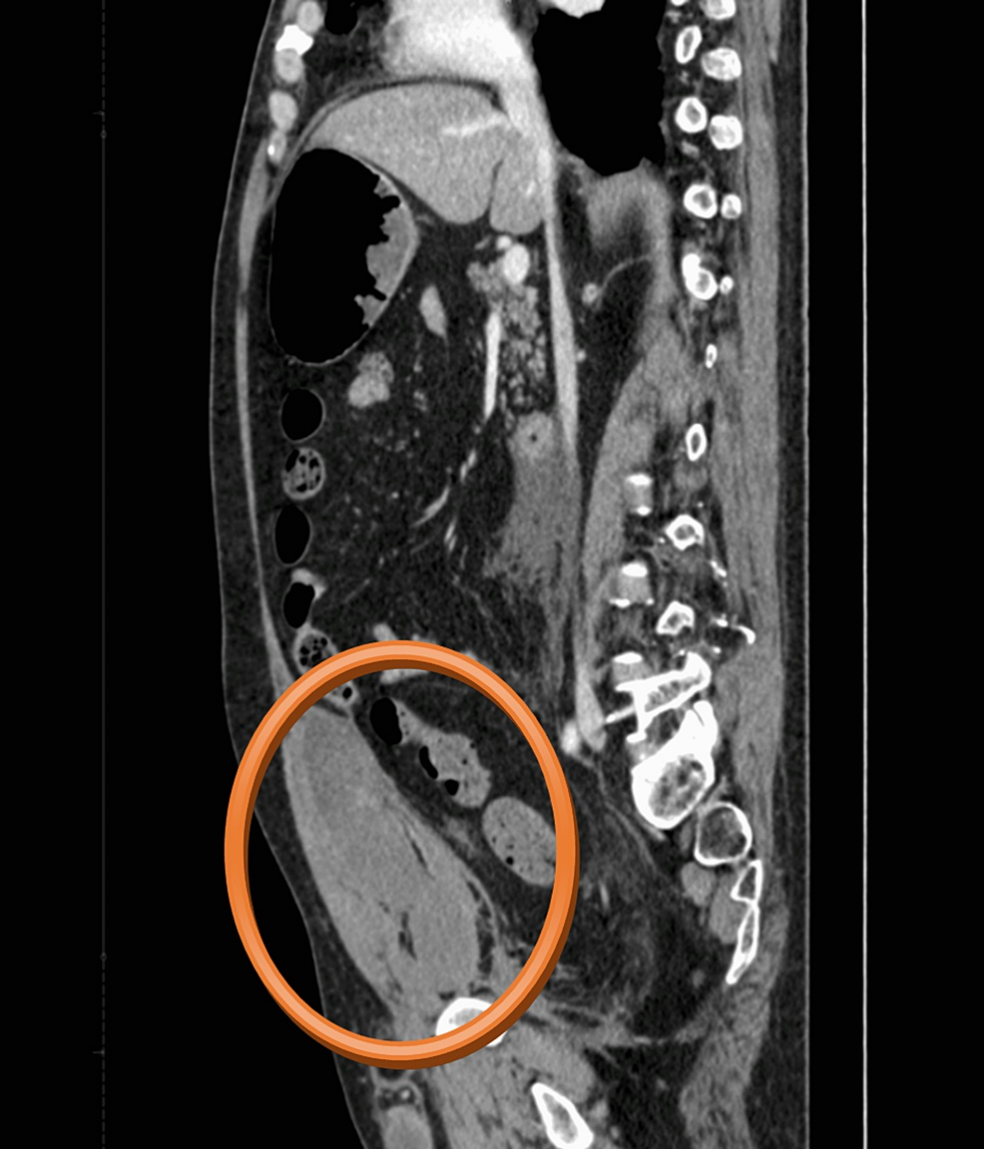
CT scan - sagittal plane Hematoma of size 130 x 80 x 50 mm in the right musculus rectus abdominis can be seen

**Figure 5:**
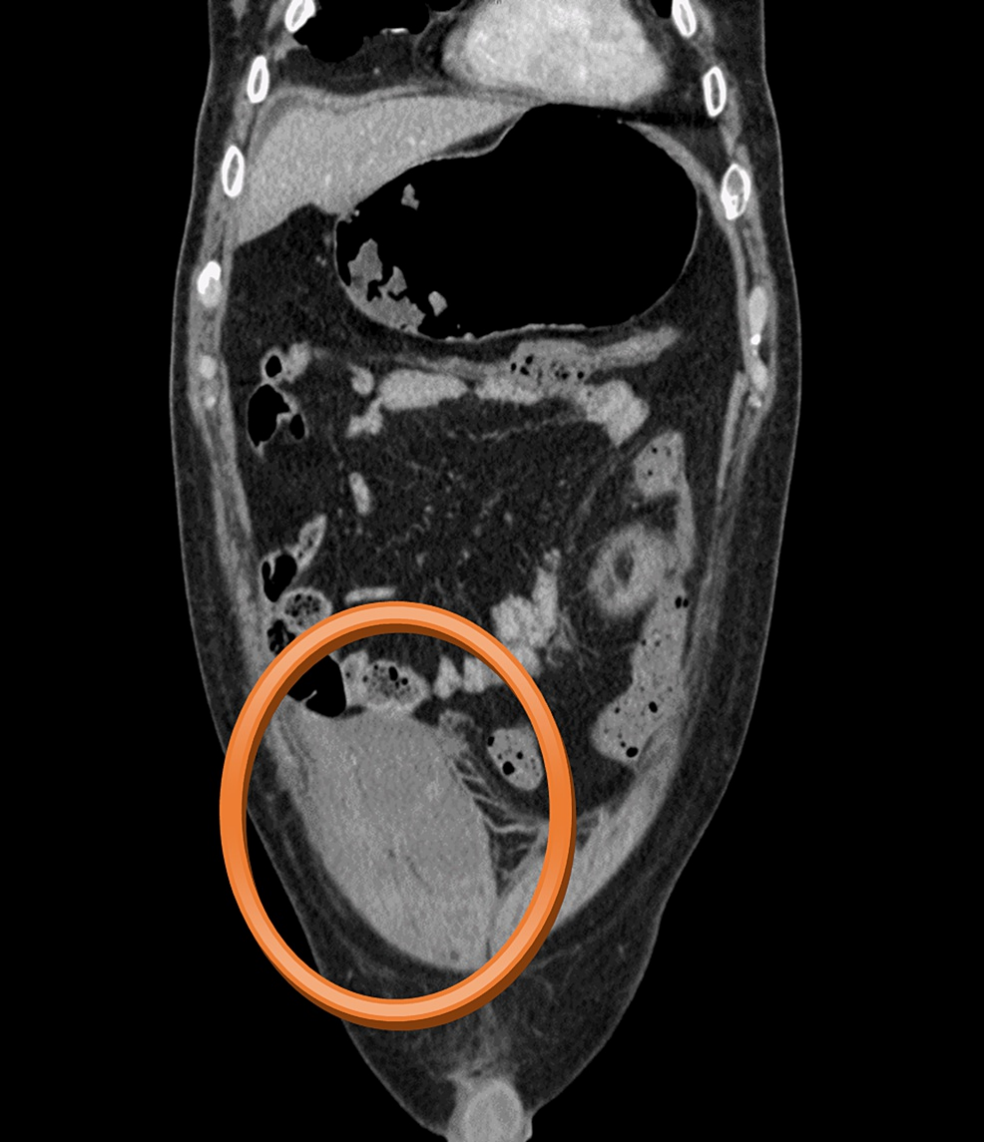
CT scan - coronal plane Hematoma of size 130 x 80 x 50 mm in the right musculus rectus abdominis can be seen

Similar fluid formation of size 130 x 80 x 50 mm was also present in the right musculus rectus abdominis, with active bleeding also present (Figures 4, 5). We decided on an immediate surgical revision. The surgery was performed in biosafety level 3 (BSL-3). We started the surgery 150 minutes after the CT examination.

We opened the abdomen by midline laparotomy prolonged to both sides. When entering the abdomen cavity, a massive hematoma outpoured from the right musculus rectus abdominis (400 mL). Immediate asystole occurred, followed by cardiopulmonary resuscitation with a 15-minute external heart massage. A definite source of bleeding was not identified. The diffuse bleeding was present in the retroperitoneal area. We proceeded with hemostasis accompanied by hot surgical cloths, and then we performed the Mikulicz tamponade of the left retroperitoneal space with five surgical cloths. We inserted a silicone drain into the pouch of Douglas and sutured the abdominal cavity by anatomical levels.

The duration of the surgery was 1 hour, and overall perioperative blood loss was measured to be approximately 2,500 mL. After the surgery, the patient was transferred to the ICU of the anesthesiology department, where the therapy continued. The patient still needed vasopressor support with noradrenaline, supplementing the circulating volume with crystalloids and blood derivatives.

Overall, the patient received 6x blood transfusions, 7x fresh frozen plasma, and 6g of fibrinogen. Protamine and tranexamic acid were given for antagonization of LMWH. Bicarbonates were applied due to severe metabolic acidosis.

Despite using all the support of circulation, the patient faced multiple organ failure, his respiratory parameters worsened, and aggressive ventilation support was incorporated. Persisting anuria and mixed acidosis continued and worsened as well.

The patient passed away 10 hours after the surgery.

## Discussion

The pandemic of more than two years has created challenges in every part of the society and medical profession is no exception to that. COVID-19 may appear in a severe, complicated form, and as shown by Al-Ani et al., Huang et al., and Chen et al. [4-6], it creates a thromboembolic situation in patients, independent of their age and comorbidities [15]. Thus, anticoagulant therapies are usually added to these patients, which is also recommended in international guidelines [16]. In our hospital, the therapeutic strategy is to reach the therapeutic dose of LWMH therapy, which seems beneficial to the majority of the patients. However, as presented in this case, a life-threatening condition may develop.

Yeoh et al. [17] showed that diagnosis of retroperitoneal hematoma requires a high degree of clinical suspicion as patients do not exhibit any clinically apparent signs and symptoms until a substantial amount of blood loss has occurred. Due to high doses of anticoagulants, frequently together with low platelets levels, these patients are at a high risk of bleeding. Yeoh et al. [17] recommend suspecting it in patients with significant groin, flank, abdominal, back pain, or hemodynamic instability after an interventional procedure or in anticoagulated patients. As shown by Kalayci [11,12], the ideal and straightforward diagnostic method is to check the hemoglobin level regularly. A contrast-enhanced CT scan of the abdomen remains the imaging modality of choice for early detection and prompt intervention [17]. As showed in our case report, COVID-19 patients treated with anticoagulants are at a risk of developing a spontaneous retroperitoneal hematoma. Although rare, it should remain a probable cause of bleeding, especially when patients present with flank pain, anemia, and signs of hypovolemia. Close monitoring and early intervention only can improve the outcome in this group of patients.

## Conclusions

The challenging era of the COVID-19 pandemic has brought many challenges to all healthcare professionals, including our surgical department, which was reprofiled to the COVID-19 department. We usually treated patients with COVID-19 bronchopneumonia in this ward; in minor cases, surgical patients with COVID-19 as an accompanying diagnosis were hospitalized in this ward. Rarely, we cured COVID-19 patients with a surgical complication such as severe bleeding into the retroperitoneal space. In our patient, there was no history of the previous injury, and clinical signs were non-specific. The patient complained of abdominal pain; and objective signs were hypotension, tachycardia, and anemia. The only possible life-saving method is a quick diagnosis and follow-up surgery. Prevention could be optimalization of anticoagulant therapy. Although treatment of LMWH in patients with severe COVID-19 bronchopneumonia is necessary, a high risk of spontaneous bleeding should be considered at therapeutic doses. Clinicians need to be aware of the high bleeding risk, which can be challenging to treat or manage, even surgically. They need to consider this as a likely cause or differential in case of unexpected anemia and non-specific abdominal symptoms. Indication for an immediate imaging examination is the only way to get us to the correct diagnosis.
